# MUCOSAL PATCH ON ANUS: A RARE SEQUEL OF SODOMY

**DOI:** 10.4103/0019-5154.44796

**Published:** 2008

**Authors:** Sudip Das, Chinmoy Kar, Parag prasun Giri

**Affiliations:** *From the Department of Dermatology and Venereology, AIIMS, New Delhi and Nilratan Sarkar Medical College and Hospital, Kolkata-700 014, India*; 1 *From the Department of Dermatology, Venereology and Leprosy, Nilratan Sarkar Medical College and Hospital, Kolkata-700 014, India*

**Keywords:** *Anus*, *mucosal patch*, *sodomy*, *syphilis*

## Abstract

A case of mucosal patch on the perianal area of a 15-year-old boy with history of frequent sodomy is presented here.

## Introduction

Mucosal patch is an important manifestation of secondary syphilis. These can be small, superficial, ulcerated or large plaques occurring on oral cavity or genital mucosa.^1^ When the maculopapular lesions involve the mucous membrane, they are called *mucous patches*, which consist of oval lesions with raised borders, central erosions, and a grey membranous covering.^2^ *Mucous patches* occur on lips, cheek, tongue, palate, tonsils, larynx and pharynx. They also occur on genital mucous membranes including those of uvula, vaginal outlet, posterior commissure, glans penis and prepuce.^2^ They are sometimes seen in anal region, in anal mucosa.^3^

Homosexuality, although legalized, now a days and people call it MSM (Men having sex with Men) and what has been a dangerous trend in our community people are using young boys, children as an object of sodomy and spreading the STDs in general and more importantly harboring the dangerous trend of infecting with HIV infection.

## Case Report

**Fig. 1:**
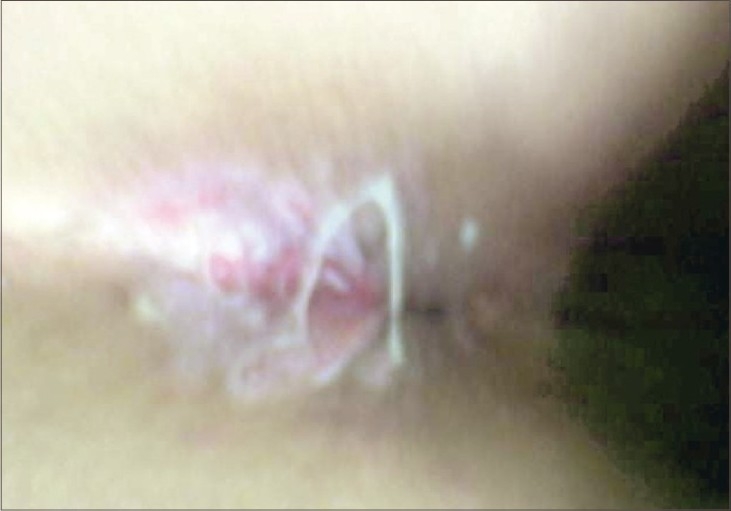
Anal mucosal patch

A 15-year-old boy, who used to massage his clients in and around Sealdah station, in Kolkata, reported to us with a reddish discoloration on anal area and also rash on palms and soles. Examination revealed mucosal patch on perianal mucosa (Fig. 1). On questioning, he revealed that he is a receptive partner to homosexual acts of his clients who pay him for this and has one to two clients per day on average.

His VDRL was positive in 1:64 titre and his TPHA was positive. His ELISA for HIV was negative. He was counseled, given injection Benzathine Penicillin 2.4 million units deep IM and he responded with resolution of clinical lesion by 2-3 weeks. His VDRL titre declines to 1:4 in 4 weeks.

## Discussion

We report the case as a unique pattern of clinical presentation with mucosal patch on perianal mucosa, which should always be examined and patient will not be forthcoming. Besides, the society needs to educate such children to refrain from such activities that may rob them of a healthy lifestyle.
